# Coupled Plasma Filtration and Adsorption (CPFA): A Single Center Experience

**DOI:** 10.5812/numonthly.11904

**Published:** 2013-09-15

**Authors:** Rizna Abdul Cader, Halim Abdul Gafor, Rozita Mohd, Wei Yen Kong, Norazimah Arshad, Norella Kong

**Affiliations:** 1 Nephrology Unit, Department of Internal Medicine, Universiti Kebangsaan Malaysia Medical Centre, Kuala Lumpur, Malaysia

**Keywords:** Plasma Filtration, Mortality, Sepsis

## Abstract

**Background:**

Coupled plasma filtration adsorption (CPFA) is a novel extracorporeal blood purification therapy for sepsis which adsorbs both proinflammatory and anti-inflammatory mediators from filtered plasma, thereby achieving early haemodynamic stability and a reduction in inotropic support requirement.

**Objectives:**

The main objective was to review our centers' experience with CPFA in septic patients.

**Patients and Methods:**

A retrospective chart review of all septic patients who received CPFA was performed. All patients were initially treated according to the ‘surviving sepsis care bundle’ with fluid resuscitation, antibiotics, and inotropes as required. CPFA was started as soon as possible after a nephrologists’ assessment.

**Results:**

Twenty five patients with sepsis received CPFA (15 M, 10 F, mean age 49.60 ± 18.97 years). Comorbidities included hypertension (n = 10, 40%), diabetes mellitus (n = 6, 24%), ischemic heart disease (n = 6, 24%), and an immunosuppressed state (n = 10, 40%). All patients received one cycle of CPFA with median duration of 5 (1-10) hours. CPFA was well tolerated but we encountered technical problems, especially filter clotting as CPFA was performed heparin free. 14 (56%) patients died within 28 days of treatment. CRP correlated with PCT (P = 0.040) and had an inverse trend with albumin (P = 0.066). Serum albumin was a strong predictor of mortality.

**Conclusions:**

The high prevalence of fungaemia and mortality could be attributed to many patients on chronic immunosuppressive therapy. Nonetheless, CPFA albeit expensive, does add to our armamentarium of extracorporeal treatment for severe sepsis. Regional citrate anticoagulation with CPFA may overcome problems with filter clotting.

## 1. Background

Sepsis is characterized by a generalized inflammatory systemic response which leads to a surge in both proinflammatory and anti-inflammatory mediators (1, 2). Interleukin (IL) 6 and tumor necrosis factor α are proinflammatory mediators and their effects include tissue injury resulting in vital organ dysfunction (3). Anti-inflammatory cytokines such as IL-4 and IL-10 counter regulate the effect of proinflammatory mediators (4). Sepsis is associated with a high morbidity and mortality which can be reduced by early diagnosis and treatment (5). Acute kidney injury is common in severe sepsis and septic shock, hence the involvement of nephrologists (6).

Coupled plasma filtration adsorption (CPFA) is a novel extracorporeal blood purification therapy for sepsis which adsorbs both proinflammatory and anti-inflammatory mediators during sepsis nonselectively (7-9). In vitro studies have demonstrated the efficacy of CPFA in adsorbing inflammatory mediators like IL-1β, IL-6, IL-8, IL-10, and tumor necrosis factor α amongst others (10). CPFA has been shown to enhance early hemodynamic stability, reduce inotropic support requirement, and improve the immune response in septic patients (7, 11, 12). However, these trials have so far failed to demonstrate any improvement in hard clinical outcomes.

**Figure 1.**
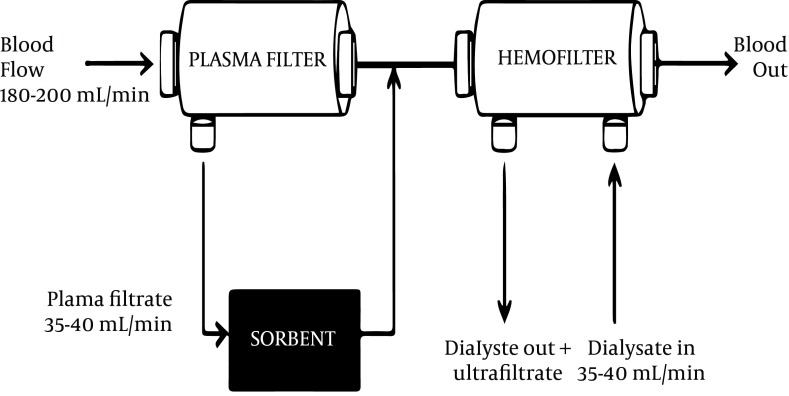
CPFA Circuit With Continuous Renal Replacement Therapy

CPFA consists of filtration, adsorption and hemofiltration. During the filtration phase, plasma is separated from blood using a plasma filter. This separated plasma then passes through a sorbent cartridge where a specific resin allows nonspecific adsorption of pro and anti-inflammatory mediators and endotoxins (13, 14). Adsorption is the accumulation of molecules on the surface of a sorbent material depending on the membrane material, pH, ionic strength and pore size (10, 13, 15). There is no contact of red blood cells, white blood cells and platelets with the sorbent, thereby preventing treatment induced thrombocytopenia. The plasma filtrate is regenerated and returned to combine with blood thereby avoiding unwanted losses. CPFA can be used with a hemofilter for additional blood purification and removal of excess fluid in the presence of acute kidney injury (Figure 1). 

Continuous renal replacement therapy is effective in reducing circulating cytokine levels compared to hemodialysis and has been proposed as a therapeutic option in sepsis (16, 17). Continuous renal replacement therapy eliminates an appreciable amount of tumor necrosis factor α and other proinflammatory cytokines (18). However, CPFA has been noted to remove more inflammatory mediators than continuous renal replacement therapy (8). High volume hemofiltration has also been demonstrated to be beneficial in sepsis with an improvement in inflammatory mediators and reduced inotropic support (19, 20).

Although studies have demonstrated CPFA to improve hemodynamic stability in septic patients, data is limited as most studies involve a very small number of patients. CPFA has been shown to be superior to high volume hemofiltration in septic patients with multiple organ dysfunction syndromes (21, 22). One study compared CPFA directly with high volume hemofiltration in twenty four critically ill patients and found CPFA to be superior in increasing the ratios of anti-inflammatory to proinflammatory mediators and restoring the leukocyte responsiveness to lipopolysaccharide (22). Another study that compared CPFA with high volume hemofiltration in eight patients with septic shock could only demonstrate a trend towards improvement in mean arterial pressure and vasopressor score in the CPFA group (23). CPFA reduced mortality rates in a rabbit model of endotoxin-mediated shock but there are no mortality studies in humans to date (9).

## 2. Objectives

At our center, continuous veno-venous hemofiltration (CVVH) and high volume hemofiltration are routinely used for septic patients with acute kidney injury. CPFA was introduced to our institution in late 2010 and was used predominantly as an additional therapy for sepsis. The main objective of this study was to review our centers experience with CPFA in septic patients.

## 3. Patients and Methods

We retrospectively reviewed the charts of all patients who received CPFA treatment during the first 18 months of its introduction at universiti Kebangsaan Malaysia medical centre (March 2011 to September 2012). We only included septic patients in this retrospective review. 

### 3.1. Patients

The inclusion criteria included patients above 18 years whom received CPFA for sepsis. Patients either had to have severe sepsis or septic shock with/without acute kidney injury. The criterion for severe sepsis was a modified version by Bone et al. and adopted by the American college of chest physicians/society of critical care medicine consensus conference (24). Patients with more than double inotropes were not considered for CPFA. We excluded patients who received CPFA for reasons other than sepsis e.g. thyroid storm. All patients were treated according to ‘surviving sepsis care bundle’ with fluid resuscitation, antibiotics, and inotropes as required by the referring teams. All these patients were referred to nephrologists for either acute kidney injury or metabolic acidosis due to sepsis. CPFA was started as soon as possible after the nephrologists’ assessment. Written informed consent for the vascular catheter and CPFA +/- CVVH was obtained from either the patient or the next of kin. Demographic and laboratory data were retrospectively collected. Outcomes were reviewed at 28 days post treatment.

### 3.2. Coupled Plasma Filtration and Adsorption

CPFA was performed using the HF440 device (Infomed, Geneva, Switzerland) with a blood flow rate of 180 - 200 mL/min, ultrafiltration rate of 35 mL/kg/h, and plasma flow rate was 20% of blood flow rate (35 – 40 mL/min). The plasma filter made of polyethersulfone had a surface area of 0.45 m² (Plasmafilter LF-050, Infomed, Geneva, Switzerland) and the sorbent cartridge was made of styrene resin with macroporous structure (Sorbent SO-310 Infomed, Geneva, Switzerland). CPFA duration was until all the resin was adsorbed on the sorbent and at this point the CPFA pump was turned off and patients continued with hemofiltration on the same device. We did not replace the sorbent after it was fully adsorbed. The hemofilter had a surface area of 1.4 m² (Hemofilter DF-140, Infomed, Geneva, Switzerland) and the prescribed ultrafiltrate flow rate of 35 mL/kg/h with a blood flow rate of 180 – 200 mL/min. Ultrafiltration rate depended on the fluid status of the patient. CVVH was performed using bicarbonate replacement fluid and the hemofilter was not routinely changed within 24 hours, unless it clotted.

A dual lumen 12F vascular catheter (Medcomp, Harleysville, USA) was inserted under ultrasound guidance using the Seldinger technique in the internal jugular vein or femoral vein. As no anticoagulant was used, 0.9% normal saline flushing using 100 mL was done at 30 minute intervals.

### 3.3. Statistical Analysis

Data was analyzed using the SPSS version 20.0 (SPSS Inc. Chicago, IL, USA). All data was tested for normality. Normally distributed numerical data were expressed as mean ± standard deviation. Non-normally distributed data were reported as median with inter-quartile range (IQR). Student’s independent and paired t-test were used for parametric data and Mann–Whitney U-test for nonparametric data to compare demographics between survivors and nonsurvivors. We used Pearson and Spearman Rho correlation to assess correlation between various parameters.

## 4. Results

A total of 25 patients with sepsis received CPFA and their demographics were tabulated in Table 1. The median duration of CPFA was 5 (1-10) hours. All patients were treated with one cycle of CPFA. After adsorption, hemofiltration was performed using the same device and 24/25 (96%) received CVVH. Platelet count at the end of treatment was 65 × 10 ^9^ (7-521).

**Table 1.** Demographics of Patients Receiving CPFA

	Mean (%)	Std Deviation (IQR)
**Gender (n)**		
Male	15 (60)	
Female	10 (40)	
**Race (n)**		
Malay	11 (44)	
Chinese	11 (44)	
Indian	3 (12)	
**Co-morbidities (n)**		
Diabetes	6 (24)	
Hypertension	10 (40)	
Ischemic heart disease	6 (24)	
Immunosuppression	10 (40)	
**Age (y)**	49.60	18.97
**Haemoglobin (g/dL)**	10.1	2.4
**White cell count (x 10^9^)**	15.6	9.5
**Platelet (x 10^9^)**	98	(8-477)
**Serum urea (mmol/L)**	16.5	8.7
**Serum creatinine (µmol/L)**	252.5	142.6
**eGFR, MDRD^a^ (mL/min/1.73 m^2^)**	27	(6-108)
**Serum albumin (g/L)**	22.32	5.17
**CRP ^a^ (mg/dL)**	19.44	15.06
**PCT ^a^ (ng/dL)**	45.42	38.13

^a^ Abbreviation: CRP; capsular reactive protein, eGFR MDRD; estimated glomerular filtration rate using modification of diet in renal disease, PCT; Procalcitonin

The causative organisms for sepsis were gram positive (n = 4, 16%), gram negative (n = 4, 16%), mixed gram positive and negative (n = 3, 12%), tuberculosis (n = 3, 12%), viral (n = 2, 8%), fungal (n = 4, 16%) and culture negative (n = 5, 20%) organisms respectively. Pneumonia (n = 12, 48%) accounted for the main cause of sepsis in these patients. This was followed by abdominal sepsis including intra-abdominal abscesses (n = 6, 24%) and unknown (n = 4, 16%). Other causes included dengue hemorrhagic shock (n = 1, 4%), meningitis (n = 1, 4%), and cellulitis (n = 1, 4%).

Most patients tolerated CPFA well but we encountered technical problems, especially plasma filter clotting as CPFA was performed heparin free. Fourteen (56%) died within 28 days of treatment. 48% of the overall cohort had concomitant disseminated intravascular coagulation (DIVC) with severe sepsis and 75% of those with DIVC died. The demographics of survivors and non survivors are shown on Table 2. We found that a low serum albumin was a predictor of mortality. 

**Table 2.** Demographics of Survivors and Non Survivors

Demographics	Survivors (n = 11)	Non Survivors (n = 14)	P value
**Age (y)**	52.45 ± 18.67	47.36 ± 19.59	NS
**Gender, No.(%)**			
Male	7/15 (46.7)	8/15 (53.3)	NS
Female	4/10 (40)	6/10 (60)	
**Diabetes, No.(%)**	3/6 (50)	3/6 (50)	NS
**Immunosuppressed, No.(%)**	5/11 (45.4)	4/14 (28.8)	NS
**DIVC, No.(%)** ^**a**^			0.071
Yes	3 (25)	9 (75)	
No	8 (61.5)	5 (38.5)	
**Serum creatinine (µmol/L)**	281 ± 181.4	230.1 ± 104.9	NS
**Serum albumin (g/L)**	25.45 ± 4.87	19.86 ± 4.03	0.005
**Hemoglobin (g/dL)**	9.6 ± 1.5	10.5 ± 2.9	NS
**WCC^**a**^ (x 10^9^)**	16.4 ± 11.2	15.0 ± 8.3	NS
**CRP (mg/dL)** ^**a**^	17.28 ± 15.67	22.42 ± 14.66	NS
**PCT, No.(%)** ^**a**^ ** (ng/dL)**	54.5 ± 38.9	37.6 ± 38.6	NS
**Duration of CPFA (h)**	5 (1-10)	3.5 (1.5-10)	NS

^a^ Abbreviation: CRP; capsular reactive protein, DIVC; disseminated intravascular coagulation, PCT; procalcitonin, WCC; white cell count

We noted a strong correlation between serum procalcitonin (PCT) and CRP (r^2^ = 0.689, P = 0.040). We also observed an inverse trend of serum CRP with albumin (r^2^ = -0.430, P = 0.066). We could not demonstrate any correlation between CRP/PCT and outcome but found a strong inverse correlation between serum albumin and outcome (r^2^ = - 0.549, P = 0.005).

## 5. Discussion

Although CPFA demonstrated an improvement in hemodynamic stability in septic patients, there are no hard clinical endpoints to date. Outcome in sepsis depends on interplay between a multitude of factors rather than a single intervention. Hence, reasons why they are no studies at present demonstrating an improvement in sepsis mortality with CPFA. However, one has to agree there is enough evidence demonstrating early hemodynamic stability in sepsis with CPFA (7, 11, 12). Lipopolysaccharide is the main component in gram negative bacterial cell wall and is involved in mediating the endotoxic shock of gram negative sepsis (13). CPFA has been shown to restore the leukocyte responsiveness to lipopolysaccharide, thereby reducing the inflammatory effects (7, 8, 22). This may partially explain how CPFA improves the hemodynamic stability, especially in gram negative sepsis. Non selective adsorption of both pro and anti-inflammatory mediators during CPFA reduces these mediators in the circulation; hence there is less vasodilatation of blood vessels enhancing hemodynamic stability.

Our cohort was septic as evidenced by high markers of sepsis (PCT and CRP). One study observed an increase in the mean arterial pressure, cardiac index, systemic venous resistance index, and a 72% decline in CRP in patients with septic shock treated with CPFA (15). The commonest cause of sepsis in our patients was pneumonia which is consistent with other reports (25, 26).

Our mortality rate in this septic cohort with acute kidney injury was high at 56% but is in keeping with the literature; whereby, the presence of acute kidney injury independently increases mortality in sepsis (27). There was a significant number (40%) of immunosuppressed patients in our cohort who were on treatment for either lupus nephritis or renal transplant recipients under nephrology follow up. One patient had acquired immunodeficiency syndrome (AIDS). The high mortality rate can also be attributed to the high number of immunosuppressed hosts. Fungal infection accounted for 16% in our cohort which is higher than the reported literature but these results are skewed by the increased number of immunosuppressed hosts. Fungal infection in immunosuppressed hosts is common and carries a grave mortality (28).

There are several studies showing the correlation between PCT and morbidity and mortality (29-31). We demonstrated a strong correlation between CRP and PCT but could not demonstrate any correlation between PCT and outcome. Being a retrospective study, not all patients had a serum PCT measurement (only 52%) prior to CPFA treatment and being a small cohort, it was difficult to demonstrate PCT correlation with outcome. Serum albumin is strongly associated with the severity and outcome of sepsis in critically ill patients and is in keeping with our findings (32, 33). We demonstrated that patients with a lower serum albumin had a higher mortality.

CPFA was well tolerated and appeared safe with no treatment-related adverse complications or hypersensitivity reaction. One patient died during the CPFA treatment but this was due to severe dengue hemorrhagic shock and DIVC rather than the effect of CPFA. Thrombocytopenia is not expected as blood cells do not come into contact with the adsorber directly. We found a significant reduction in platelet count at 24 hours after CPFA (P = 0.009). However, thrombocytopenia could be due to a multitude of factors including severe sepsis and DIVC. When we reanalyzed the platelet count by groups, we found that those with DIVC had a significant reduction in their platelet count (P = 0.011); whereas, those without DIVC had no reduction in platelet count (P = 0.221). We can therefore conclude that CPFA treatment does not cause thrombocytopenia per se but rather other factors like DIVC.

The main technical problem that we did encounter was plasma filter clotting. Nearly half of these patients’ with sepsis also had DIVC; therefore, CPFA was performed heparin free. Although the rest did not have DIVC, they had some coagulation abnormalities, thrombocytopenia or recent surgical intervention therefore requiring heparin free CPFA. Despite regular saline flushing, we encountered problems with plasma filter clotting as early as two hours. The reason for the clotting could be due to the high concentration of coagulation factors like plasma prothrombin, D dimer and thrombin which are produced in response to activation of the extrinsic coagulation pathway in severe sepsis (34). Simultaneously, there are low levels of protein C and antithrombin III which also contribute to thrombosis in sepsis (35). However, persistent thrombin activation and fibrin formation with insufficient thrombin neutralization leads to DIVC in severe sepsis. Some of the ways to overcome this clotting problem include using heparin as an anticoagulant. However as mentioned before, this was not possible in a large amount of patients due to DIVC. Another way to overcome the clotting problem is through the use of regional citrate anticoagulation (36). Citrate has been used as an anticoagulant in the extracorporeal circuit with calcium being infused prior to the venous blood return to the patient (37). Regional citrate anticoagulation requires two extra infusions pumps for citrate and calcium and regular monitoring of serum calcium to avoid hypocalcaemia (37). It has been suggested that the addition of another filter (cascade filter) prior to the sorbent cartridge may alleviate the problems with clotting by removing the coagulation factors in the extracorporeal circuit.

Outcomes in this cohort are not good due to several reasons; CPFA was performed quite late rather than early in sepsis. This is because patients’ were only referred to nephrologists after they developed established acute kidney injury or metabolic acidosis. This particular cohort not only suffered from severe sepsis but also acute kidney injury which increased their morbidity and mortality.

The strength of our study lies in the fact that this is the largest number of patients receiving CPFA. The main drawback in our study was the cost of new sorbents. Even though previous studies have been promising, they did not take cost into account for each treatment. Due to the prohibitive costs of CPFA disposables, we could only afford to provide a single cycle of CPFA followed by CVVH per treatment; whereas, most studies provided about 4 to 8 cycles of CPFA per treatment session of 24 hours.

Conclusion: We have demonstrated that CPFA is a safe and well tolerated procedure which may have a role in sepsis especially if instituted early. Although most studies have performed several cycles of CPFA, we have demonstrated that even a single cycle of CPFA may add to our armamentarium of treatment for sepsis. Filter clotting is a problem with CPFA which can be overcome by regional citrate anticoagulation or the use of a cascade filter. 
